# Immunological Effects and Viral Gene Expression Determine the Efficacy of Oncolytic Measles Vaccines Encoding IL-12 or IL-15 Agonists

**DOI:** 10.3390/v11100914

**Published:** 2019-10-03

**Authors:** Paul S. Backhaus, Rūta Veinalde, Laura Hartmann, Jessica E. Dunder, Lara M. Jeworowski, Jessica Albert, Birgit Hoyler, Tanja Poth, Dirk Jäger, Guy Ungerechts, Christine E. Engeland

**Affiliations:** 1 National Center for Tumor Diseases, Im Neuenheimer Feld 460, 69120 Heidelberg, Germany; paul.backhaus@web.de (P.S.B.); ruta.veinalde@biomed.lu.lv (R.V.); laura.hartmann@dkfz-heidelberg.de (L.H.); jessica.dunder@nct-heidelberg.de (J.E.D.); lara.jeworowski@ewetel.net (L.M.J.); jessica.albert@nct-heidelberg.de (J.A.); birgit.hoyler@nct-heidelberg.de (B.H.); dirk.jaeger@nct-heidelberg.de (D.J.); guy.ungerechts@nct-heidelberg.de (G.U.); 2 Clinical Cooperation Unit Virotherapy, German Cancer Research Center, 69120 Heidelberg, Germany; 3 Medical Faculty, University of Heidelberg, 69120 Heidelberg, Germany; 4 German Cancer Research Center, 69120 Heidelberg, Germany; 5 Faculty of Biosciences, University of Heidelberg, 69120 Heidelberg, Germany; 6 Department of Medical Oncology, University Hospital Heidelberg, 69120 Heidelberg, Germany; 7 CMCP—Center for Model System and Comparative Pathology, Institute of Pathology, University Hospital Heidelberg, 69120 Heidelberg, Germany; tanja.poth@med.uni-heidelberg.de; 8 Center for Cancer Therapeutics, Ottawa Hospital Research Institute, Ottawa, ON K1H 8L6, Canada; 9 Research Group Mechanisms of Oncolytic Immunotherapy, Clinical Cooperation Unit Virotherapy, German Cancer Research Center, 69120 Heidelberg, Germany

**Keywords:** cancer immunotherapy, oncolytic virus, measles virus, interleukin-12, interleukin-15

## Abstract

Tumor-targeted immunomodulation using oncolytic viral vectors is currently being investigated as a promising strategy in cancer therapy. In a previous study, we showed that a measles virus Schwarz vaccine strain (MeVac) vector encoding an interleukin-12 fusion protein (FmIL-12) is an effective immunotherapy in the MC38cea murine colon adenocarcinoma model. We hypothesized that MeVac encoding interleukin-15 may mediate enhanced T and NK cell responses and thus increase the therapeutic efficacy, especially in NK cell-controlled tumors. Therefore, we generated MeVac vectors encoding an interleukin-15 superagonist, FmIL-15. Replication and oncolytic capacity, transgene expression, and functionality of MeVac FmIL-15 vectors were validated in vitro. Effects on the tumor immune landscape and therapeutic efficacy of both FmIL-12 and FmIL-15 vectors were studied in the MC38cea and B16hCD46 tumor models. Treatment with MeVac FmIL-15 increased T and NK cell infiltration in both models. However, MeVac FmIL-12 showed more robust viral gene expression and immune activation, resulting in superior anti-tumor efficacy. Based on these results, MeVac encoding a human IL-12 fusion protein was developed for future clinical translation.

## 1. Introduction

Oncolytic viruses (OVs) selectively replicate in malignant cells, leading to tumor cell lysis. Since viral replication and thus viral gene expression are confined to the malignant tissue, oncolytic viruses can be used as vectors for the delivery of therapeutic genes to the tumor site. Such therapeutic genes can include prodrug convertases, radiosensitizers, proapoptotic, or immunomodulatory molecules [1]. The latter concept is based on the fact that tumor cell death, in the context of viral infection, is highly immunogenic, due to the release of tumor antigens in concert with danger-associated and pathogen-associated molecular patterns (DAMPs and PAMPs). This can serve as an in situ tumor vaccine that elicits an anti-tumor immune response. Accordingly, oncolytic vectors, which express immunomodulatory transgenes, have been developed to enhance this response and to serve as tumor-targeted immunotherapy [2]. Most prominently, granulocyte–macrophage colony stimulating factor (GM-CSF) has been encoded in several oncolytic viruses, including talimogene laherparepvec (T-VEC), the first oncolytic virus approved by the FDA and EMA for the treatment of advanced melanoma [3]. Several other cytokines have also been tested, including interleukin-2 (IL-2), tumor necrosis factor-α (TNFα), and interleukin-12 (IL-12). Notably, tumor-restricted expression, by means of an oncolytic vector, enables tumor immunomodulation with agents that can be highly toxic when administered systemically.

Oncolytic viruses, derived from the measles virus (MV) vaccine strains, have been tested in numerous preclinical studies and have advanced to clinical trials [4]. Measles virus is a negative-strand RNA virus of the *Paramyxoviridae* family. A versatile reverse genetics system is available which allows for the design of genetically modified vectors with enhanced tumor selectivity and therapeutic activity [5]. Measles vaccine strain viruses mainly enter cells via the complement regulatory protein CD46, which is often overexpressed in malignant cells [6]. Retargeting to a cell surface molecules of choice can be achieved by mutating the receptor binding sites within the viral attachment protein hemagglutinin and fusing a targeting moiety of choice to its C-terminus [7]. Moreover, targeting on the post-entry level can be achieved by using microRNA target sites, which exploits the fact that, in cancer cells, microRNA expression profiles are frequently altered in comparison to healthy tissue [8]. Furthermore, vectors with additional transcription units have been developed, which enable the insertion of additional genes as reporters or for therapeutic purposes [5,7,9,10].

MV vectors have been utilized for targeted tumor immunomodulation [11,12,13,14,15,16,17]. We previously developed oncolytic MV vectors derived from the Schwarz vaccine strain (MeVac), which encode different immunomodulators: An IL-12 fusion protein (FmIL-12), GM-CSF, IP-10, anti-CTLA-4, anti-PD-L1, and CD80 [18]. In the MC38cea tumor model, a colorectal adenocarcinoma syngeneic to fully immunocompetent C57BL/6 mice, the MeVac vector encoding FmIL-12 (MeVac FmIL-12) showed highest therapeutic efficacy [18]. Treatment with MeVac FmIL-12 led to 90% complete tumor remissions, protective anti-tumor immune memory, and was associated with an increase in tumor-infiltrating activated cytotoxic T cells and a Th1 cytokine profile. However, despite high levels of CD69 expression in intratumoral NK cells, their abundance was low. We concluded that this may result from activation-induced cell death (AICD). Since some tumors, e.g., the B16 murine melanoma model, are known to be mainly controlled by NK cell activity [19], we assumed that MeVac FmIL-12 might not be effective against such tumors. Rather, we reasoned that an interleukin-15 (IL-15) agonist may be beneficial, since IL-15 is known to promote both CD8+ and NK cell proliferation and activation, while simultaneously counteracting AICD [20,21,22]. Furthermore, IL-15 does not support regulatory T cell function, but promotes T cell memory formation [23,24]. These properties designate IL-15 as a candidate for cancer immunotherapy.

IL-15 mainly acts in its membrane-bound form via trans-presentation by the IL-15 receptor alpha (IL-15Rα) to effector cells expressing the IL-2R/15Rβ-γc [25,26]. Activation of these receptors initiates JAK1/JAK3 and STAT3/STAT5, as well as PI3K/Akt, Shc, and ERK1/2 signaling [26,27]. While soluble IL-15 has a short half-life and low biological activity, IL-15/IL-Rα complexes are recycled endosomally and can mediate sustained signaling activity [28]. Several IL-15 agonists, comprising IL-15 and the IL-15Rα, have previously been developed and showed enhanced bioactivity compared to IL-15 alone [29,30,31]. For this study, we selected an IL-15 agonist consisting of the IL-15Rα sushi domain and IL-15, joined by a glycine-serine linker, as described by Mortier et al. [31].

MeVac variants encoding a murine version of the IL-15/IL-15Rα fusion protein (FmIL-15) were cloned and their immunomodulatory and anti-tumor activity was compared to MeVac FmIL-12 in two immunocompetent mouse models of measles oncolysis. While we found an increase in tumor-infiltrating activated T and NK cells after treatment with MeVac FmIL-15, MeVac FmIL-12 proved to be superior in terms of tumor control and survival. Analyses of tumor specimens suggested that sustained viral gene expression and immune cell effector activity are underlying mechanisms. These results prompted us to develop MeVac encoding a human IL-12 fusion protein (FhIL-12) for future clinical application.

## 2. Materials and Methods

### 2.1. Cell Lines and Viruses

Vero African green monkey kidney cells (CCL-81) and B16 murine melanoma cells (CRL-6322) were obtained from the American Type Culture Collection. The Vero-αHis cell line, stably expressing a single-chain antibody against the His_6_ tag [32], was a gift from S.J. Russell (Mayo Clinic, Rochester, MN, USA). MC38cea murine colon adenocarcinoma cells, stably expressing human carcinoembryonic antigen (CEA) [33] and the parental MC38 cell line, were a gift from R. Cattaneo (Mayo Clinic, Rochester, MN, USA). B16hCD46 cells were generated by lentiviral transduction as described previously [17]. The mouse cytotoxic T lymphocyte cell line CTLL-2 was obtained from H.-J. Delecluse (DKFZ Heidelberg). COLO 205 cells were obtained from A. Jassowicz (DKFZ Heidelberg), and DLD-1 and HCT 116 were a gift from S. Fröhling (DKFZ Heidelberg), and HT29 as well as KM12 cells were obtained from C. Plass (DKFZ Heidelberg).

Vero, MC38, HCT 116, and KM12 cell lines were cultivated in Dulbecco’s modified Eagle’s medium (DMEM) (Thermo Fisher, Waltham, MA, USA) and were supplemented with 10% fetal bovine serum (FBS) (PAN-Biotech, Aidenbach, Germany). B16hCD46, COLO 205, DLD-1, and HT29 cells were cultivated in Roswell Park Memorial Institute (RPMI) 1640 medium (Thermo Fisher) with 10% FBS. CTLL-2 cells were maintained in RPMI + 10% FBS and 50 U/mL recombinant murine interleukin-2 (rmIL-2, Miltenyi Biotech, Bergisch Gladbach, Germany). All cell lines were maintained at 37 °C in a humidified atmosphere with 5% CO_2_ and were routinely tested for mycoplasma contamination.

For the generation of recombinant measles viruses, the reverse genetics system, originally described by Radecke et al. [34] and adapted for RNA polymerase II [35], was used. Plasmids encoding measles Schwarz/Moraten vaccine strain anti-genomes (pcMeVac)based on this system have been reported previously [18].

### 2.2. Cloning and Rescue of Recombinant MeVac

Cloning of pcMeVac P FmIL-12, pcMeVac ld EGFP (encoding enhanced green fluorescent protein), pcMeVac H IgG1-Fc (encoding the Fc region of human immunoglobulin G type 1), and their CEA-targeted variants have been described previously [18]. To generate pcMeVac encoding FmIL-15, a cDNA fragment encoding a murine IL-15/IL-15Rα fusion construct, based on the sequence published by Tosic et al. [36], was designed. The cDNA fragment comprised a Kozak sequence, the mouse immunoglobulin kappa light chain leader sequence as secretion signal, the N-terminal sushi domain of IL-15Rα, a GG(SGG)_6_ linker, and mIL-15 lacking the signal peptide and propeptide flanked by *MluI* and *PauI* restriction sites for insertion into additional transcription units in the MeVac genome. The cDNA sequence was optimized for *Mus musculus* codon usage with GENEius software (Biolink Informationstechnologie GmbH, Martinsried, Germany) and was obtained by gene synthesis (Eurofins, Ebersberg, Germany). The FmIL-15 cassette was inserted into additional transcription units (ATUs) downstream of the measles virus leader or *P* open reading frame, respectively, to obtain pcMeVac ld FmIL-15 and pcMeVac P FmIL-15. For retargeting to CEA, the *SpeI-SpeI* fragment was exchanged for the *SpeI-SpeI* fragment of pcMeVac Hbl-αCEA [18].

To generate MeVac FhIL-12 vectors, cDNA from human peripheral blood mononuclear cells (PBMCs) was amplified using the following primers (all obtained from Eurofins):

FhIL-12 p40 for   5′ CCCGGGACGCGTGCCACCATGTGTCACCAGCAGT 3′
FhIL-12 p40 rev  5′ AGATCCGCCGCCACCGCCACCACTGCAGGGCAC 3′
FhIL-12 p35 for   5′ GGTGGCGGTGGCGGCGGATCTAGAAACCTCCCC 3′
FhIL-12 p35 rev  5′ CCCGGGGCGCGCTCACTAGGAAGCATTCAGATAGCTC 3′

The resulting p40-linker and linker-∆p35 fragments were then subjected to fusion PCR with flanking primers—FhIL-12 p40, which includes an *MluI* restriction site and FhIL-12 p35 rev that includes a *PauI* restriction site as well as an additional stop codon to comply with the rule of six [37]. The complete FhIL-12 cassette was then inserted into ATUs downstream of the *P* or *H* open reading frame, respectively, to obtain MeVac P FhIL-12 and MeVac H FhIL-12.

To rescue recombinant viruses, 5 µg of the respective pcMeVac anti-genomic plasmid, 500 ng pCG N, 500 ng pCG L, and 100 ng pCG P plasmids were transfected into Vero (untargeted MeVac variants) or Vero-αHis cells (targeted variants) in a six-well format in DMEM + 2% FBS + 50 µg/mL Kanamycin using FuGENE^®^ HD Transfection Reagent (Promega, Madison, WI, USA). When syncytia had formed, indicating viral replication, cells were scraped and used for further virus propagation.

### 2.3. MeVac Titration, Propagation and Infection Experiments

The methods for propagation and titration of recombinant measles viruses have been described in detail [38]. In brief, viral titers were determined by serial dilution of virus stocks on Vero or Vero-αHis cells in DMEM + 10% FBS in 96-well plates. Titers in cell infectious units (ciu) per mL were calculated by multiplying the number of syncytia counted at 48 or 72 h with the respective dilution factor. For propagation, Vero or Vero-αHis cells were inoculated with measles variants at a multiplicity of infection (MOI) of 0.03 in OptiMEM. When syncytia had spread across the entire cell layer, cells were scraped, vortexed, and frozen in liquid nitrogen. After thawing, cell debris was removed by centrifugation (5 min at 2500× *g* and 4 °C) and aliquots of virus stocks were stored at −80°C until further use.

For infection experiments, cells were seeded one day prior to inoculation with MeVac at the indicated MOI. Virus inoculation was performed in OptiMEM for two hours before continuing cultivation in cell line-specific medium.

### 2.4. Transgene Expression and Bioactivity

FmIL-15 expression was assessed by analyzing supernatants from MC38cea cells infected with MeVac FmIL-15 cells using the Mouse IL-15/IL-15R Complex ELISA Ready-SET-Go Kit (eBioscience, Schwerte, Germany). To test FmIL-15 activity, CTLL-2 cells were starved from rmIL-2 for 4 h and were subsequently re-stimulated with RPMI + 10% FBS containing diluted supernatants from MeVac-infected cells, 50 U/mL rmIL-2 as positive control, or medium only as negative control. After 48 h, cell viability was assessed using the Colorimetric Cell Viability Kit III (XTT, PromoKine, Heidelberg, Germany), according to the manufacturer’s protocol. Western blot analysis was performed with total cell lysates in RIPA buffer and rabbit polyclonal Stat5-reactive antisera L-20 and N-20 (Santa Cruz Biotechnology, Dallas, TX, USA), monoclonal rabbit anti- Phospho-Stat5 (Tyr694) (C11C5) antibody (Cell Signaling Technology, Danvers, MA, USA), and secondary goat anti-rabbit-IgG, HRP-coupled (Bethyl Laboratories, Montgomery, TX, USA), all at a dilution of 1:10,000, as well as POD-coupled mouse anti-β-actin (Sigma-Aldrich; 1:20,000, St. Louis, MO, USA).

FhIL-12 levels in supernatants of infected Vero cells were quantified using the human IL-12 p70 ELISA Read-SET-Go Kit (eBioscience) according to the manufacturer’s instructions.

### 2.5. Characterization of MeVac Variants

The methods for characterization of viral replication and cytotoxicity have been described in detail [38]. To generate one-step growth curves, 1 × 10^5^ cells in 12-well plates were infected at MOI 3, scraped at designated timepoints, and progeny titers were determined by titration. Direct oncolytic activity was assessed by performing XTT assay at designated timepoints after infection of 2 × 10^5^ cells in 6-well plates at MOI 3.

### 2.6. Animal Experiments

All animal experimental procedures were approved by the regional council (Regierungspräsidium Karlsruhe, AZ 35-9185.81/G-58/17, 06-27-2017) and were conducted in accordance with the German Animal Protection Law and institutional guidelines.

Six to eight week old female C57BL/6J mice were obtained from Harlan Laboratories, Rossdorf, Germany. Mice were housed under SPF conditions in groups of five mice in individually ventilated cages in the Center for Preclinical Research of the German Cancer Center (DKFZ).

1 × 10^6^ tumor cells in PBS were injected subcutaneously into the right flank and treatment was initiated when the tumor volume reached 50–100 mm^3^. For flow cytometry experiments, mice with smaller tumors were allocated to Mock or MeVac H IgG1-Fc treatment to prevent drop-out of mice in control groups. For survival experiments, mice were stratified into treatment groups to achieve an equal distribution of tumor volumes. Investigators were blinded to treatments. Mice received intratumoral (i.t.) injections of 5 × 10^5^ (MC38cea) or 1 × 10^6^ (B16hCD46) ciu of the respective virus diluted in OptiMEM to 100 μL total volume on four consecutive days. A dose of 5 × 10^5^ ciu was applied in the MC38cea model due to difficulties in generating high-titer virus stocks with CEA-retargeted vectors. Mice in the Mock group received treatment with 100 µL OptiMEM. Tumor volumes were determined three times a week by caliper measurements; tumor volumes were calculated using the following formula: Largest diameter × (smallest diameter)^2^ × 0.5.

In efficacy experiments, mice were sacrificed when tumor volume reached 1000 mm^3^, tumor largest diameter reached 15 mm, or when tumor ulceration occurred. Tumor rechallenge experiments were carried out four months after MeVac treatment. 1 × 10^5^ parental MC38 cells suspended in 100 μL PBS were injected subcutaneously into the left flank and mice were monitored, as described above. For histological assessment, formalin-fixed, paraffin-embedded tumor samples were stained with hematoxylin/eosin and analyzed by a veterinary pathologist (T.P.) who was blinded to treatment.

### 2.7. Flow Cytometry

Flow cytometry analyses were carried out with single cell suspensions prepared from freshly explanted tumors. Tumors were minced with a scalpel. MC38cea tumors were digested for 30 min at 37 °C in RPMI + 5 % FBS + 200 IU/mL Collagenase type I (Thermo Scientific) with vigorous shaking every 10 min. Minced B16hCD46 and digested MC38cea tumor samples were passed through 100 µm cell strainers and cells were pelleted by centrifugation (5 min at 300× *g*) and counted. 2 × 10^6^ cells were used for staining with the following antibodies (all from BD Biosciences, Heidelberg, Germany): PerCP-Cy5.5 Anti-Mouse CD45.2 (clone 104) 1:500, Alexa Fluor^®^ 700 Anti-Mouse CD3 Molecular Complex (clone 17A2), 1:500, APC-Cy7 Anti-Mouse CD4 (clone GK1.5), 1:500, APC Anti-Mouse CD8a (clone 53–6.7), 1:500, FITC Anti-Mouse CD335 (NKp46) (clone 29A1.4), 1:400, and PE Anti-Mouse CD69 (Clone H1.2F3), 1:250. DAPI staining was used for dead cell exclusion. Data were acquired on a BD FACS LSR II instrument with FACS Diva software (version 8.0.1, BD Biosciences) and analyzed with FlowJo software (version X10.0.7r2, Tree Star Inc., Ashland, OR, USA). Only samples with at least 1000 cells were included in the analysis.

### 2.8. RT-qPCR

Total RNA was extracted from infected cells and samples of explanted tumors were preserved in RNAlater (Qiagen, Hilden, Germany) using the RNeasy Mini Kit (Qiagen) according to the manufacturer’s instructions. cDNA synthesis was performed using the Maxima H Minus First Strand cDNA Synthesis Kit (Thermo Scientific) with 1 µg RNA and oligo(dT)_18_ primers according to the manufacturer’s protocol.

RT-qPCR reactions were prepared with 1 μL cDNA, 200 nM respective forward and reverse primers, and 10 μL Power SYBR^®^ Green PCR Master mix (Thermo Scientific) in a total volume of 20 μL. The following target-specific primers were used (all obtained from Eurofins):

mBcl2 for    5′–AGGCTGGGATGCCTTTGTGG–3′,
mBcl2 rev      5′–ACTTGTGGCCCAGGTATGC–3′,
mCish for      5′–CCTCGTCCTTCCAAGCTGTT–3′,
mCish rev      5′–CCCAGTACCACCCCCTGTA–3′,
mFasL for      5′–TCTGTGGCTACCGGTGGTAT–3′,
mFasL rev     5′–GTACTGGGGTTGGCTCACG–3′,
mGzmb for    5′–ACAAAGGCAGGGGAGATCAT–3′,
mGzmb rev   5′–CGAATAAGGAAGCCCCCACA–3′,
mL13A for     5′–GGCTGCCGAAGATGGCGGAG–3′,
mL13A rev    5′–GCCTTCACAGCGTACGACCACC–3′.
MV-N 241 for  5′-TTACCACTCGATCCAGACTTC-3′,
MV-N 331+ rev   5′-CCTATTAGTGCCCCTGTTAGTTT-3′,
FmIL-12p40 for   5′–CACTCCCCATTCCTACTTCT–3′,
FmIL-12p35 rev  5′–CAGGATGCAGAGCTTCATTT–3′,
FmIL-15 for     5′–CAACAGGGGACACAACCTGT–3′,
FmIL-15 rev     5′–TGCACTTGAGGCTAGGTGTG–3′.

Primers for FmIL-12 and FmIL-15 were designed to only amplify the fusion proteins encoded in MeVac and not endogenous IL-12, IL-15, or IL-15Rα. PCR was carried out with the following conditions: 10 min initial denaturation at 95 °C, 40 cycles of 10 s denaturation at 95 °C, 60 s annealing and extension at 60 °C (*Bcl-2,* *Cish, Fasl, Gzmb, FmIL12,* and *L13a*), 62 °C (MV *N*), or 65 °C (*FmIL-15)* and 5 s fluorescence detection at 78 °C using a C1000 Touch Thermal Cycler (CFX96 Real-Time System, and Bio-Rad, Hercules, CA, USA). Data were acquired using Bio-Rad CFX Maestro 1.1 software (Bio-Rad). Melting curve analysis was performed to identify specific amplification. Minus reverse transcriptase controls and no template controls were run in parallel with cDNA samples.

### 2.9. NanoString Gene Expression Profiling and Pathway Analysis

Total tumor RNA was used for NanoString gene expression analysis, which was performed by the nCounter Core Facility Heidelberg. Quality control (QC) was carried out using an Agilent Bioanalyzer 2100 and Qubit system. 25 ng RNA from samples that passed QC were hybridized with the CodeSet Immunology Panel (Mouse; NanoString Technologies, Seattle, WA, USA) and processed using nCounter SPRINT Profiler. Raw data was normalized to the set of internal reference genes included in the CodeSet panel. Data was analyzed using nSolver 4.0 software and the Advanced Analysis package (NanoString Technologies). Differential gene expression data was analyzed using Ingenuity Pathway Analysis (IPA, Qiagen, www.qiagen.com/ingenuity). IPA core analysis and comparison analysis were performed. Differentially activated canonical pathways and diseases and functions were evaluated, and comparison results were displayed as heat maps.

### 2.10. Statistical Analyses

Statistical analyses were performed using GraphPad Prism software (version 6.01; GraphPad Software, LaJolla, CA, USA). Tumor volume distribution, ELISA, RT-qPCR, and flow cytometry data were analyzed by unpaired *t*-test or one-way ANOVA (Kruskal-Wallis) with Dunn’s multiple comparison post-hoc test. Multiplicity adjusted *p* values are reported for data analyzed with ANOVA. *p* values below 0.05 are considered statistically significant. Survival curves were analyzed by log-rank (Mantel–Cox) test with Bonferroni correction for multiple comparisons. Results are considered statistically significant if the *p* value is lower than the corrected threshold after Bonferroni correction.

## 3. Results

### 3.1. Oncolytic Measles Vaccines Encoding FmIL-12 or FmIL-15

We previously generated MeVac FmIL-12, an oncolytic MV Schwarz vector encoding a murine IL-12 fusion protein. This fusion protein consists of the murine IL-12 p40 and p35 subunits separated by a glycine-serine linker, as described by Lieschke et al. [39]. FmIL-12 was inserted into an additional transcription unit downstream of the *P* open reading frame. This position was chosen on the basis of previous analyses of MV Edmonston B vaccine strain vectors. In these experiments, FmIL-12 was inserted at three different positions within the viral genome, downstream of the leader and *P* and *H* open reading frames, respectively. The P FmIL-12 variant stood out in terms of replication, cytopathic effects, and FmIL-12 expression after infection [40].

**Figure 1:**
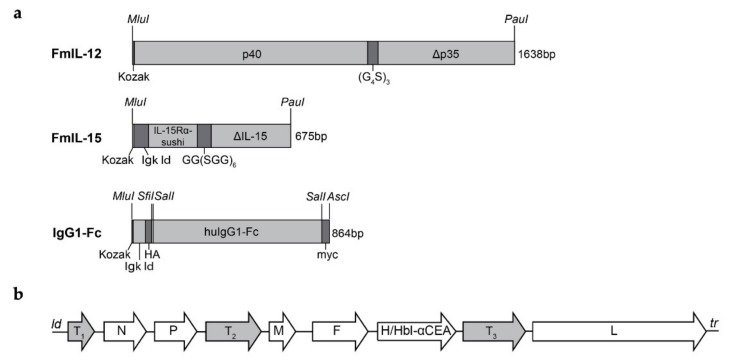
Cloning of oncolytic measles vaccines encoding FmIL-12 or FmIL-15. (**a**) Design of the FmIL-12, FmIL-15, and IgG1-Fc expression cassettes. The FmIL-12 cassette consists of the mIL-12 p40 and p35 subunits separated by a (G_4_S)_3_ linker preceded by a Kozak sequence. The signal peptide sequence was deleted from the p35 subunit (Δp35). The FmIL-15 cassette consists of a Kozak sequence, sequences encoding the immunoglobulin kappa light chain leader (Igκ ld), the mIL-15Rα sushi domain, a GG(SGG)_6_ polypeptide linker, and mIL-15 lacking the signal peptide and propeptide (ΔmIL-15). Flanking *MluI* and *PauI* restriction sites were added for insertion into the MeVac genome. The IgG1-Fc cassette in control vectors includes Kozak and Igκ ld sequences, HA and myc tags, and the Fc region of human immunoglobulin G type 1 flanked by *MluI* and *AscI* restriction sites. (**b**) Genome schematic. The FmIL-12 expression cassette was inserted into an additional transcription unit downstream of the MeVac *P* open reading frame (position T_2_). The FmIL-15 expression cassette was inserted downstream of the MeVac ld or *P* open reading frame, respectively (positions T_1_ or T_2_). The IgG1-Fc cassette was inserted downstream of the *H* open reading frame (position T_3_). Retargeting to carcinoembryonic antigen (CEA) was achieved by replacing hemagglutinin (H) with an H variant harboring mutated receptor-binding sites and a CEA-specific scFv fused to its C-terminus (Hbl-αCEA). N, P, M, F, H, and L: Nucleocapsid, phosphoprotein, matrix, fusion, hemagglutinin, and large (polymerase) open reading frames. *ld*, *tr*: leader and trailer.

MeVac vectors encoding a murine IL-15 superagonist, FmIL-15, were cloned using the IL-15/IL-15R fusion protein sequence published by Tosic et al. [36]. The fusion protein encompasses IL-15 linked to the IL-15Rα-sushi domain by a glycine-serine polypeptide. In addition, preceding Kozak and Igκ leader sequences were included to facilitate translation and secretion. This FmIL-15 cassette was inserted into an additional transcription unit downstream of the MeVac leader or *P* open reading frame, respectively. Vectors encoding the Fc region of human IgG1 (IgG1-Fc) were used as controls, as in our previous study [18]. Since we aimed to investigate these vectors in the MC38cea model, the MeVac attachment protein hemagglutinin was retargeted to CEA using an antibody single-chain variable fragment, as described previously [18]. For investigations in the B16hCD46 model, MeVac variants with native tropism, that is, human CD46-tropic, were used. Schematics of the transgene cassettes and the recombinant MeVac genome are depicted in Figure 1a,b, respectively.

### 3.2. Secretion and Bioactivity of FmIL-15

**Figure 2:**
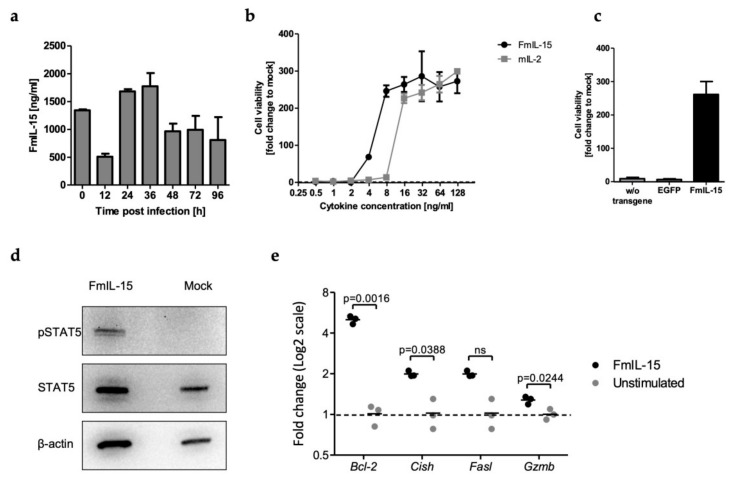
Secretion and bioactivity of MeVac-encoded FmIL-15. (**a**) MC38cea cells were inoculated with MeVac ld FmIL-15 Hbl-αCEA at a multiplicity of infection (MOI) of 3 in triplicates. FmIL-15 levels in the cell culture supernatant were determined using an ELISA for the murine IL-15/IL-15R complex between 0 and 96 h after inoculation. Mean values of triplicate infections with standard deviations are shown. (**b**) FmIL-15 was obtained from supernatants of Vero-αHis cells infected with MeVac ld FmIL-15 Hbl-αCEA. Cytokine-dependent CTLL-2 cells were starved from cytokines for 4 h and then stimulated with FmIL-15, rmIL-2 (positive control), or RPMI + 10% FCS (unstimulated). FmIL-15 and rmIL-2 were used in 2-fold serial dilutions from 125 ng/mL to 0.5 ng/mL. After 48 h, cell viability was determined with an XTT cell viability assay. Cell viability is depicted as fold change over cell viability of unstimulated CTLL-2 cells (mock, fold change = 1). Mean values of triplicate stimulations with standard deviations are shown. (**c**) Undiluted supernatants from Vero-αHis cells infected with MeVac ld FmIL-15 Hbl-αCEA (corresponding to 2500 ng/mL FmIL-15), MeVac ld EGFP (encoding enhanced green fluorescent protein) Hbl-αCEA, or MeVac Hbl-αCEA (without transgene) were used for the CTLL-2 stimulation assay, as described in (**b**). (**d**) Previously starved CTLL-2 cells were stimulated with diluted supernatants from Vero-αHis cells infected with MeVac ld FmIL-15 Hbl-αCEA (corresponding to a saturating concentration of 100 ng/mL FmIL-15) or mock stimulation (medium only). After 48 h of stimulation, phosphorylated STAT5 (pSTAT5), total STAT5, and β-actin were detected by Western blot. (**e**) Cells were treated as described in (**d**) and mRNA levels of the IL-15 target genes *B cell lymphoma 2 (Bcl-2), Cytokine-inducible SH2-containing protein (Cish), Fas ligand (Fasl),* and *Granzyme B (Gzmb)* were determined by quantitative reverse transcription PCR (RT-qPCR). Data are plotted as 2-ΔΔCp values with *mL13a* as reference gene. Data were analyzed by unpaired *t*-test. Dots representing individual samples with a line at the mean are shown.

To study expression kinetics of FmIL-15, murine tumor cells were infected with MeVac encoding FmIL-15 and cell culture supernatants were analyzed using an ELISA specific for the murine IL-15/IL-15R complex (Figure 2a). FmIL-15 was detected in the inoculum (0 hours post infection, h.p.i.), resulting from FmIL-15 expressed in Vero cells during virus production. FmIL-15 levels dropped after inoculation, but rose again at 24 h.p.i. and peaked at 36 h.p.i., indicating secretion of FmIL-15 from infected tumor cells.

Functionality of secreted FmIL-15 was tested in a lymphocyte proliferation assay with starved cytokine-dependent CTLL-2 cells. Defined concentrations of FmIL-15 from infected cell supernatants were able to provide an equally strong or stronger stimulus for lymphocyte proliferation when compared to rmIL-2 (Figure 2b). To exclude non-specific stimulation of lymphocyte proliferation by other viral or cellular components, CTLL-2 cells were cultured in supernatants of cells infected with different MeVac variants (Figure 2c). In contrast to supernatants from cells infected with MeVac ld FmIL-15 Hbl-αCEA, supernatants from cells infected with parental or EGFP-encoding MeVac did not promote survival of the cytokine-dependent lymphocytes. To demonstrate functional activity of FmIL-15 on a molecular level, cell lysates of CTLL-2 cells stimulated with FmIL-15 were probed for phosphorylated STAT5 (Figure 2d). Phosphorylated STAT5 was detected in CTLL-2 cells stimulated with FmIL-15, but not in controls, indicating activation of JAK/STAT signaling by FmIL-15. RT-qPCR was performed for several IL-15 target genes (Figure 2e). Expression levels of *Bcl-2*, *Cish,* and *Gzmb* mRNA were found to be significantly increased upon stimulation of CTLL-2 cells with FmIL-15 secreted from infected cells.

Taken together, these findings showed that FmIL-15 is expressed and secreted by murine tumor cells upon infection with FmIL-15-encoding oncolytic measles vectors and that this secreted form of the IL-15/IL-15R complex is functional, promoting lymphocyte survival and proliferation, activation of JAK/STAT signaling, and expression of downstream IL-15 target genes.

### 3.3. Replication and Cytotoxicity of Recombinant Measles Viruses in Murine Tumor Cell Lines

**Figure 3:**
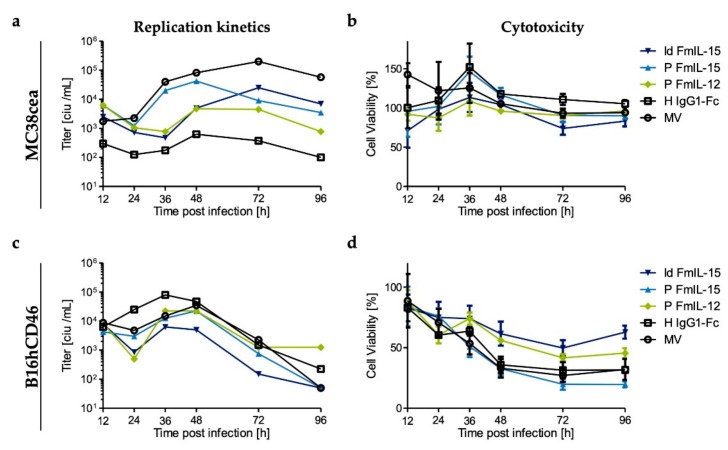
In vitro replication and cytotoxicity of MeVac variants. Murine tumor cells (MC38cea and B16hCD46) were inoculated with the indicated MeVac variants at an MOI of 3 in triplicates. (**a**,**c**) To generate one-step growth curves, cells were harvested at indicated timepoints and progeny virus titers of pooled triplicates were determined by titration assay. (**b**,**d**) XTT cell viability assays were performed at designated timepoints after infection. Viability of mock-infected cells was defined as 100%. The mean and standard deviation of octuplicate titration wells (**a**,**c**) or triplicate infections (**b**,**d**) are shown. Note that MeVac variants retargeted to CEA (Hbl-αCEA) were used for inoculation of MC38cea cells, while MeVac variants with native tropism (CD46) were used in case of B16hCD46 cells.

In this study, a murine colorectal adenocarcinoma model, MC38cea, and a murine melanoma model, B16hCD46, were used, which are both syngeneic to C57BL/6 mice. Prior to in vivo experiments, replication kinetics and cytotoxicity of the recombinant measles viruses were evaluated. Viruses used in the MC38cea model harbor a hemagglutinin variant that allows cell entry via CEA, while viruses used in the B16hCD46 model harbor unmodified hemagglutinin. To analyze growth kinetics, one-step growth curves were generated after infection of MC38cea and B16hCD46 (Figure 3a,c). It should be noted that measles virus is adapted to primate cells and viral replication is thus impaired in murine cells [41]. All viruses were clearly attenuated in the murine cells compared to primate Vero or Vero-αHis cells (Supplementary Materials Figure S1). For most viruses, titers peaked at 36 to 48 h.p.i. Direct virus-induced cytotoxicity was assessed by cell viability assay at depicted timepoints after infection (Figure 3b,d). Viability of MC38cea cells was only mildly affected by infection with the measles virus vectors, with no overt differences between the different variants. B16hCD46 appeared to be more permissive to the MV vectors. Inoculation with MeVac ld FmIL-15, the most attenuated virus in this experiment, led to a decrease in cell viability to about 50% of mock-treated cells, while infection with MeVac P FmIL-15 reduced cell viability to 20%. Direct cytotoxic effects exerted by MeVac P FmIL-12 were slightly stronger than those exerted by MeVac H IgG1-Fc and parental MV, all of which reduced cell viability to about 40% of mock infection.

The vectors harboring the FmIL-15 cassette downstream of the leader appeared to be more attenuated in comparison to MeVac P FmIL-15 (Figure 3 and Supplementary Materials Figure S1). FmIL-15 expression in the supernatant did not differ significantly at later timepoints after infection. However, the ld FmIL-15 constructs mediated increased FmIL-15 expression within the first 24 h (Supplementary Materials Figure S1). Due to these differences in replication, direct oncolysis, and transgene expression, we decided to investigate both ld FmIL-15 and P FmIL-15 vectors in vivo.

### 3.4. Immunovirotherapy with MeVac Encoding FmIL-15 Increases CD8+ T Cell and NK Cell Infiltration

**Figure 4:**
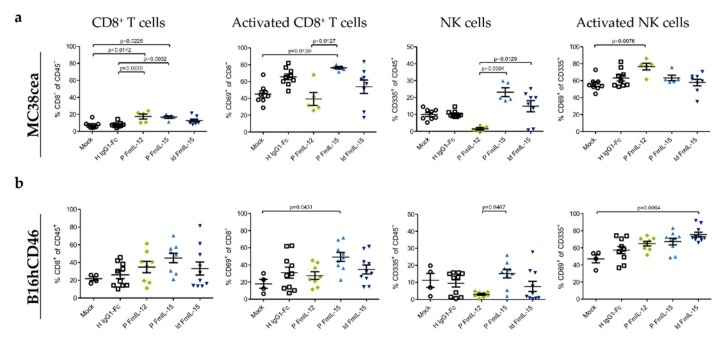
Tumor-infiltrating lymphocytes after MeVac treatment. MC38cea (**a**) or B16hCD46 (**b**) tumor cells were implanted subcutaneously in the right flank of C57BL/6J mice. When tumors had reached a size of 50–100 mm³, mice received intratumoral injections of indicated MeVac variants on four consecutive days. Flow cytometry of tumor-infiltrating lymphocytes was performed one day after the fourth treatment. Symbols representing individual animals and the group mean with standard deviation are shown.

To study the effects of treatment with immunomodulatory oncolytic MV on the composition of tumor-infiltrating lymphocytes (TILs), C57BL/6J mice bearing subcutaneous syngeneic MC38cea or B16hCD46 tumors received intratumoral injections of the different MeVac vectors and flow cytometry was performed 24 h after the last treatment. In the MC38cea model (Figure 4a), the CD8+ T lymphocyte fraction among CD45+ cells was significantly increased by local immunotherapy with MeVac P FmIL-12 Hbl-αCEA or MeVac P FmIL-15 Hbl-αCEA. Treatment with MeVac P FmIL-15 Hbl-αCEA led to a significant increase in the CD69+ fraction of CD8+ T cells, indicating increased cytotoxic T lymphocyte activation. A similar trend was seen with MeVac ld FmIL-15 Hbl-αCEA and MeVac H IgG1-Fc Hbl-αCEA. As observed in earlier experiments [18], treatment with MeVac P FmIL-12 Hbl-αCEA led to a depletion of intratumoral NK cells. NK cell abundance among CD45+ cells in tumors was significantly increased after treatment with MeVac P FmIL-15 Hbl-αCEA, in comparison to MeVac P FmIL-12 Hbl-αCEA, and a similar, but weaker effect was seen using MeVac ld FmIL-15 Hbl-αCEA. Activation of NK cells, as determined by CD69 expression, was increased in tumors treated with MeVac P FmIL-12 Hbl-αCEA, consistent with data published earlier by Veinalde et al. [18].

In the B16hCD46 model (Figure 4b), the effects on the T cell compartment were less pronounced. However, a trend towards higher CD8+ T lymphocyte abundance among CD45+ cells was seen in tumors treated with measles vectors encoding FmIL-12 or FmIL-15. CD69 expression on CD8+ T cells was increased markedly in the MeVac P FmIL-15 group, while trends towards higher CD69 expression could be observed in all MeVac groups compared to the mock group. Treatment with MeVac P FmIL-12 resulted in a striking decrease in NK cell abundance among CD45+ cells, comparable to the effect seen in the MC38cea model. NK cell abundance was not distinctly changed by vectors encoding FmIL-15. However, CD69 expression on NK cells was increased significantly after treatment with MeVac ld FmIL-15, signifying higher NK cell activation. Compared to mock treatment, a trend towards increased CD69 expression on NK cells was also observed for MeVac P FmIL-15, MeVac P FmIL-12, and MeVac H IgG1-Fc. Histological samples and analyses of four MC38cea tumor samples from each treatment group revealed tumor cell death in several MeVac treated tumors (Supplementary Materials Figure S2 and Supplementary Materials Table S1). While intratumoral lymphocyte infiltration was only observed in one tumor treated with MeVac P FmIL-15 Hbl-αCEA, all tumor samples showed a peritumoral mixed-cellular inflammatory infiltrate that tended to be more pronounced in tumors treated with MeVac FmIL-12 Hbl-αCEA (Supplementary Materials Table S1).

### 3.5. Therapeutic Efficacy in Syngeneic Mouse Models

**Figure 5:**
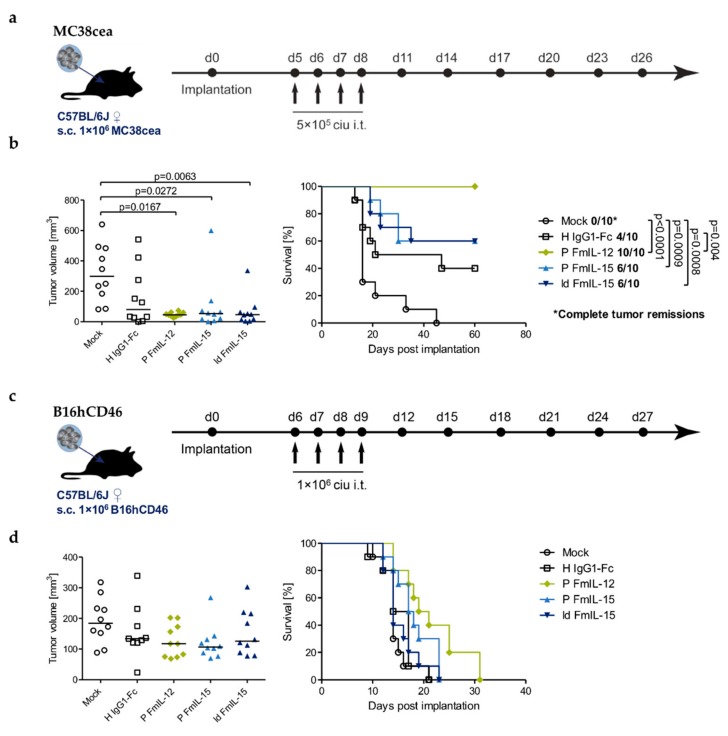
Therapeutic efficacy. C57BL/6J mice bearing s.c. MC38cea (**a**,**b**) or B16hCD46 (**c**,**d**) tumors were treated by intratumoral (i.t.) injections of 1 × 10^6^ ciu of indicated MeVac variants on four consecutive days. (**a**,**c**) Treatment schedules. (**b**,**d**) Left panels: Tumor volume distribution on day 13 (**b**) and 10 (**d**), which was the last day when all mice were alive. Values for individual animals and group median are shown. Right panels: Kaplan–Meier survival analysis.

The therapeutic efficacy of the measles vectors was assessed in both the MC38cea and the B16hCD46 model (Figure 5, Supplementary Materials Figure S3).

In the MC38cea model, treatment with MeVac H IgG1-Fc induced a complete tumor remission in four out of ten mice. MeVac ld FmIL-15 Hbl-αCEA and MeVac P FmIL-15 Hbl-αCEA induced a complete remission in six out of ten individuals. Overall, neither significantly prolonged survival compared to MeVac H IgG1-Fc Hbl-αCEA. Therapeutic efficacy was significantly increased for MeVac P FmIL-12 Hbl-αCEA, which achieved a complete remission in all ten animals (Figure 5b).

Once established, B16hCD46 tumors grew aggressively and oncolytic virotherapy with MeVac H IgG1-Fc, MeVac P FmIL-15 and MeVac ld FmIL-15 had only marginal, if any, effects on tumor progression and survival. By contrast, treatment with MeVac P FmIL-12 seemed to slightly delay tumor progression (Supplementary Materials Figure S3) and led to a minor, non-significant survival benefit (Figure 5d).

Long-term survivors from the MC38cea experiment were rechallenged with parental MC38 cells (Supplementary materials Figure S4). Parental MC38 cells were chosen to exclude the dependence on the foreign antigen human CEA. Tumor engraftment was observed in all five naïve control mice and in all mice previously treated with MeVac P FmIL-15. Interestingly, all four mice previously treated with MeVac H IgG1-Fc, six out of ten mice previously treated with MeVac P FmIL-12, and five of six mice from the MeVac ld FmIL-15 group rejected secondary tumor engraftment, indicating a systemic, protective antitumor immune response following treatment with oncolytic measles vectors.

### 3.6. Differential Expression of Viral and Immune-Related Genes In Vivo

**Figure 6:**
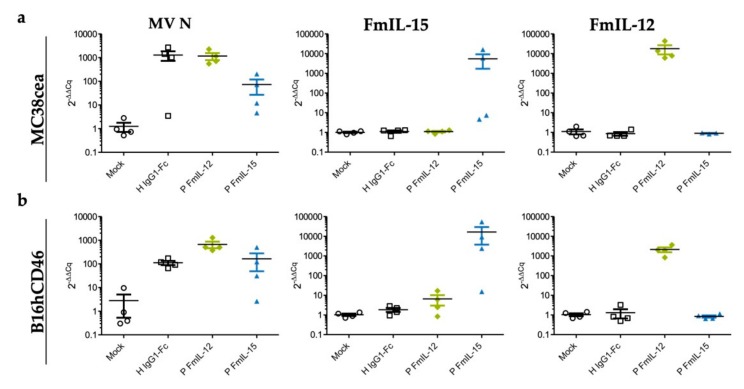
Viral gene expression in vivo. Samples (*n* = 4 per group) from MC38cea tumors (**a**) and B16hCD46 tumors (**b**) were collected 24 h after the last treatment and total RNA was extracted. RT-qPCR was performed with primers specific for MV *N*, *FmIL-15*, and *FmIL-12* with *mL13A* as reference.

Although MeVac FmIL-12 did not exhibit more efficient replication or direct oncolytic effects in vitro (Figure 3) and MeVac FmIL-15 induced a favorable immune profile in vivo (Figure 4), therapeutic efficacy of MeVac FmIL-15 was inferior to MeVac FmIL-12 (Figure 5). To understand the underlying mechanisms, we investigated the expression of viral and immune-related genes in vivo. Taking into account its overall more favorable immune profile (Figure 6) and better comparability to MeVac P FmIL-12 due to transgene position, we selected the P FmIL-15 variants for further analyses.

RNA was extracted from tumor tissue explants from the experiments shown in Figure 4. We performed RT-qPCR using primers specific for the measles virus *N* gene, *FmIL-15*, and *FmIL-12*. The expression of MV *N* was markedly lower for MeVac P FmIL-15 Hbl-αCEA compared to MeVac P FmIL-12 Hbl-αCEA and MeVac H IgG1-Fc Hbl-αCEA in the MC38cea model, indicating a lower replicative capacity of this vector. Differences were less pronounced in B16hCD46 tumors. In this model, MV *N* RNA levels in tumors injected with MeVac H IgG1-Fc were the lowest, followed by MeVac P FmIL-15 and then MeVac P FmIL-12. As expected, the expression of *FmIL-15* or *FmIL-12* was confined to the respective treatment group. Notably, a larger variation between both *N* and transgene RNA levels was observed in the FmIL-15 treatment groups.

**Figure 7:**
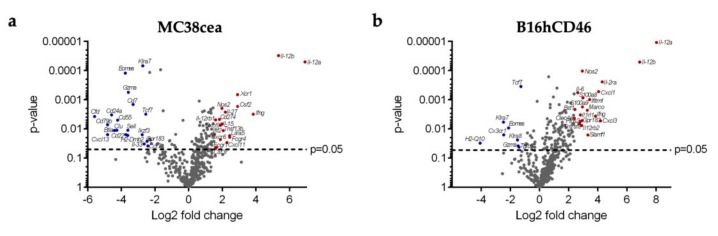
Immune expression profiling. RNA was extracted from *n* = 4 tumors from each treatment group and expression profiling was carried out using the NanoString Mouse Immunology Panel. Volcano plots show differential gene expression between the FmIL-15 and FmIL-12 treatment groups in the (**a**) MC38cea and (**b**) B16hCD46 tumor models. Each dot represents one gene. The top 20 significantly upregulated genes in the FmIL-12 versus the FmIL-15 group (Log2 Fold change >1 and *p*-value < 0.05) are shown in red. The top 20 significantly downregulated genes in the FmIL-12 versus the FmIL-15 group (Log2 Fold change <−1 and *p*-value < 0.05), i.e., upregulated in the MeVac FmIL-15 group, are shown in blue.

To further investigate the mechanisms underlying the differing therapeutic efficacies between the FmIL-12 and FmIL-15 encoding vectors, gene expression profiling was performed with the NanoString Mouse Immunology Panel (Figure 7 and Supplementary Materials). Differential gene expression analysis for the MeVac P FmIL-12 and MeVac P FmIL-15 treatment groups is depicted in Figure 7. The top 20 genes significantly upregulated in the MeVac FmIL-12 group in both models included molecules involved in FmIL-12 signaling, e.g., *Il12a, Il12b, Ifng, Il27, Il12rb1*, *Il12rb2,* as well as T lymphocyte activation and recruitment in general, e.g., *Cxcl11, Il15,* and *Cxcl9*. The genes significantly upregulated in the MeVac FmIL-15 group in the MC38cea model included factors associated with NK and T cell activation, differentiation, and recruitment, e.g., *Ccr7, Ccl19, Tcf7*, and *Eomes*, as well as genes associated with B cell function, e.g., *Ikzf3, Cd22,* and *Cd79b*. In the B16hCD46 model, only few genes were significantly upregulated, including genes involved in T and NK cell function, e.g., *Gzma, Klra8, Klra7, Eomes,* and *H2-Q10*.

Ingenuity Pathway Analysis of the NanoString data revealed that pathways associated with immune response activation in general were upregulated in both the MeVac FmIL-12 and MeVac FmIL-15 groups in comparison to the control vector group, MeVac H IgG1-Fc, in the MC38cea and B16hCD46 models (Supplementary Materials Figure S5). However, when comparing the MeVac FmIL-15 with the MeVac FmIL-12 treatment group, there was a stronger activation of most of these pathways in the latter in both models, and especially in the B16hCD46 model. This included pathways linked to lymphocyte activation, but also dendritic cell maturation and diverse immune signaling pathways. One possible explanation for limited replication and persistence of MeVac FmIL-15 could have been an enhanced anti-viral response. Interestingly, however, the expression data show that anti-viral signaling was also more pronounced after treatment with MeVac FmIL-12. Upregulation of e.g., *Myd88*, *Irf1,* and *Stat1* was observed after treatment with MeVac FmIL-12 compared to MeVac FmIL-15. Genes linked to both anti-viral and anti-tumor immune effector responses such as toll-like receptor 8 (TLR8), tumor necrosis factor (TNF), and interferon γ (IFN γ) were upregulated after MeVac FmIL-12 treatment. Overall, these data indicate that, despite the TIL profile observed after MeVac FmIL-15 treatment, MeVac FmIL-12 induced overall stronger immune effector responses.

### 3.7. Oncolytic Measles Vaccines Encoding FhIL-12 for Treatment of Human Colorectal Cancer

**Figure 8:**
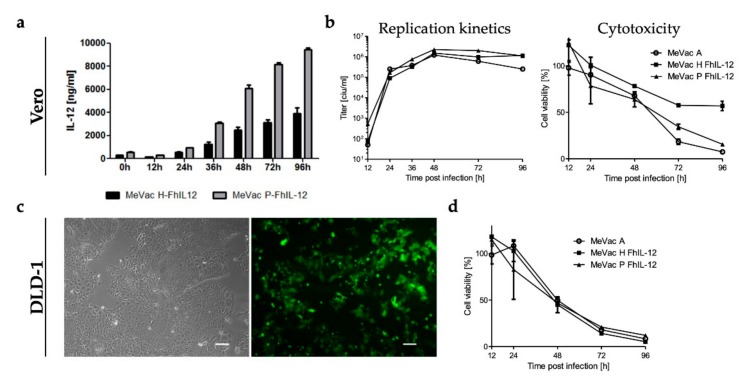
Oncolytic measles vaccine vectors encoding a human IL-12 fusion protein. (**a**) Vero cells were infected at an MOI of 3 and FhIL-12 in cell culture supernatants was quantified by ELISA. (**b**) Left panel: To generate one-step growth curves, Vero cells were infected at an MOI of 3 and viral progeny at designated timepoints were quantified by titration assay. Right panel: XTT cell viability assay was performed after infection of Vero cells at an MOI of 1. (**c**) DLD-1 human colon cancer cells were subjected to mock treatment (left panel) or inoculated with MeVac ld EGFP at an MOI of 1. Images were acquired 48 h after inoculation. Scale bars: 100 µm. (**d**) DLD-1 cells were inoculated with indicated MeVac variants at an MOI of 1 and cell viability was determined by XTT assay at designated timepoints. Cell viability data is presented as a percentage of mock (100%). Mean and standard deviation of triplicate samples are shown.

These results prompted us to generate oncolytic MeVac vectors encoding a human IL-12 fusion protein (FhIL-12) in analogy to the MeVac FmIL-12 vectors. We chose to insert the FhIL-12 cassette either downstream of the P or H open reading frames, yielding MeVac P FhIL-12 and MeVac H FhIL-12 after successful rescue. Secretion of FhIL-12 into the supernatant of infected cells was confirmed by ELISA (Figure 8a). As a result of the transcription gradient within the measles virus genome [42], infection with MeVac P FhIL-12 resulted in higher expression levels compared to MeVac H FhIL-12. In case of a potent immunostimulatory molecule such as IL-12, moderate expression levels may be beneficial to avoid both immune exhaustion and toxicity. Growth curve analyses showed that both FhIL-12 vectors have similar replication kinetics as parental MeVac and direct cytotoxicity is only slightly attenuated in primate Vero cells (Figure 8b). Based on our in vivo data in the MC38cea model, we considered colorectal cancer as a target tumor entity for MeVac FhIL-12. We tested a panel of human colorectal cancer cell lines for susceptibility to MeVac using a variant encoding EGFP to monitor viral gene expression (Figure 8c and Supplementary Materials Figure S6). All cell lines showed high rates of infection and pronounced EGFP expression already 24 h after inoculation. Further, cell viability assays revealed sensitivity of human colorectal cancer cell lines to MeVac oncolysis. As an example, cytotoxicity of parental MeVac and MeVac FhIL-12 vectors against DLD-1 cells is presented in Figure 8d.

## 4. Discussion

Interleukin-15 is currently being investigated as an immunotherapeutic agent for the treatment of cancer [43] [NCT01021059, NCT01727076, NCT01885897]. It has been shown to potently stimulate CD8+ effector T cells, memory-phenotype CD8+ T cells, and NK cells, while offering some advantages over IL-12 as it does not stimulate regulatory T cell function and inhibits the induction of AICD [20,21,22,23,24]. This effect can be enhanced by using an IL-15/IL15Rα fusion protein, which mimics the natural mode of IL-15/IL-15R interaction via trans-presentation by the IL-15Rα chain [30,44].

The measles virus Schwarz vaccine strain (MeVac) has previously been used for therapeutic gene delivery to tumors, specifically also for targeted immunomodulation [11]. We hypothesized that MeVac equipped with an IL-15/IL-15R fusion protein may have enhanced immunotherapeutic efficacy by stimulating anti-tumor CD8+ effector T cell and NK cell responses.

This study demonstrates that MeVac can be engineered to encode an IL-15/IL-15Rα fusion protein. We demonstrated that FmIL-15 is expressed by infected murine tumor cells and that the secreted fusion protein is functionally active, as shown by its ability to promote survival and to induce the expression of IL-15 target genes in cytokine-dependent lymphocytes in vitro. We studied the effects of MeVac FmIL-15 on the composition of tumor-infiltrating lymphocytes in vivo using two murine tumor models syngeneic to fully immunocompetent C57BL/6J mice, MC38cea and B16hCD46. Treatment with MeVac FmIL-15 led to enhanced CD8+ T cell and NK cell infiltration and activation, in line with our hypotheses. This has also been shown in other studies investigating oncolytic vectors encoding an IL-15/IL-15R fusion protein [36].

Therapeutic efficacy of MeVac vectors encoding FmIL-15 was evaluated in the MC38cea and B16hCD46 models and was compared to the MeVac P FmIL-12 vector developed previously. Other studies have demonstrated the efficacy of IL-15 and IL-12 based immunotherapeutics in these models. For instance, in experiments by Ugen et al., in vivo electroporation of B16 tumors with an IL-15 DNA plasmid led to durable tumor remissions in a subgroup of treated mice [45]. Kishida et al. reported delayed tumor progression after electroporation with an IL-12 plasmid [46]. In a study using the MC38 model, intraperitoneal IL-15 alone led to prolonged survival, but did not induce complete tumor regression [47]. However, combination with an anti-CD40 antibody significantly enhanced therapeutic efficacy, indicating that additional immune stimulation is required for tumor control in this model. Several studies have described combination immunotherapies including both IL-12 and IL-15 [48,49,50]. However, we are not aware of any previous systematic comparison of IL-12 and IL-15 immunotherapy in preclinical models.

Additional immune stimulation could be conveyed by oncolytic virotherapy and several oncolytic viruses encoding IL-15 agonists have been described previously. A Newcastle Disease virus (NDV) for expression of IL-15 has been studied in vitro [51]. An NDV variant encoding both IL-15 and IL-7 developed as a cancer vaccine conferred delayed outgrowth of B16 tumors [52]. A vesicular stomatitis virus (VSV) encoding IL-15 was not superior to parental VSV in a subcutaneous CT26 colon cancer model, but was more effective in preventing lung colonization, which was associated with increased peripheral NK cell counts. However, neither anti-tumor nor anti-viral CD8+ T cell responses were significantly enhanced [53]. The myxoma virus reported by Tosic et al. prolonged survival in B16 tumors in both Rag-/- and C57BL/6 mice compared to the parental vector [36]. However, no durable remissions were observed. A vaccinia virus encoding an IL-15 superagonist demonstrated CD8+-dependent efficacy in an intraperitoneal MC38 model, leading to complete remissions in a high percentage of treated mice [54].

In the present study, the effect on tumor-infiltrating lymphocyte composition did not translate into a significant improvement in survival and MeVac P FmIL-12 was superior in both tumor models. Possible underlying mechanisms are manifold, as mechanisms of action of immunovirotherapy include factors spanning from the oncolytic properties of the vector within the tumor microenvironment to local and systemic anti-tumor and anti-viral immune responses.

The oncolytic efficacy of the vector is determined by its replicative and lytic capacities. These can be affected by vector design, and in the case of *Paramyxoviridae,* specifically by the position of the transgene within the viral genome, which also holds true for the MeVac FmIL-15 constructs. Insertion of the FmIL-15 transgene downstream of *P* had less impact on replication and cytotoxicity than the insertion of the transgene downstream of the leader (Supplementary Materials Figure S1). However, the in vitro comparison of the vectors used in the animal experiments (Figure 3) cannot explain the superior efficacy of MeVac P FmIL-12, which neither showed increased viral progeny nor increased direct cytotoxicity compared to MeVac P FmIL-15. Notably, we found that intratumoral levels of viral *N* gene expression after treatment with MeVac P FmIL-12 and MeVac P FmIL-15 (Figure 6) differed markedly, especially in the MC38cea model, indicating that in vivo replication kinetics may not be reflected by in vitro growth curve analyses. Moreover, viral gene expression levels were more heterogeneous after treatment with MeVac FmIL-15, which may indicate less robust vector properties. Thus, vector design, and consequently oncolytic properties, may be one factor contributing to the superior efficacy of the FmIL-12 vectors.

However, murine tumors show only limited permissiveness for measles virus. Therefore, the role of replication and direct oncolysis in murine tumor models is debatable. Previous studies have evaluated the role of viral replication in the context of measles immunovirotherapy. Speck and Heidbuechel et al. demonstrated comparable efficacy of UV-inactivated MV and replicating MV against B16 tumors, suggesting that viral replication is not a major determinant of efficacy in this tumor model [17]. In contrast, Grossardt et al. found less effective anti-tumor T cell priming with UV-inactivated MV GM-CSF in the MC38cea model [13]. It must be noted, however, that these studies used the MV Edmonston B vaccine strain, which differs in terms of lytic and immunomodulatory properties from the Schwarz strain used in the present study [55].

Aside from the virus strain, vector-mediated immunomodulation is determined by the expression kinetics of the immunomodulatory transgene, which, again, is closely linked to viral replication. Previous experiments by Veinalde et al. showed increasing levels of FmIL-12 to above 2000 ng/mL FmIL-12 in cell culture supernatants after inoculation of MC38cea cells with MeVac P FmIL-12 [18]. In contrast, inoculation with MeVac P FmIL-15 resulted in stable levels of approximately 5000 pg/mL FmIL-15 (Supplementary Materials Figure S1), which may result from both de novo expression as well as endosomal recycling. Direct comparison of FmIL-15 and FmIL-12 levels is challenging, as both cytokines have different effective concentrations. Regardless, these data may indicate limited bioavailability of FmIL-15 after infection of murine tumor cells, which may in turn limit immune effector responses.

Effects of MeVac FmIL-15 and MeVac FmIL-12 on the tumor immune environment were investigated in the MC38cea and B16hCD46 models of MV immunovirotherapy. Notably, the baseline immune landscapes of MC38- and B16-derived tumors differ substantially. MC38 is immunogenic, characterized by a dense immune infiltrate and a high mutational burden, while B16 is an immune-deserted tumor with a scarce immune infiltrate and a high degree of immunosuppression [56]. Nevertheless, changes in TIL composition were similar in both tumor models: Treatment with MeVac FmIL-15 led to an increase in CD8+ T cell and NK cell infiltration in the MC38cea model. Additionally, CD8+ T cells were highly activated, as shown by the upregulation of CD69. A trend towards higher CD8+ T cell infiltration and activation, as well as increased NK cell activation was observed in the B16hCD46 model after treatment with MeVac FmIL-15. MeVac P FmIL-12 treatment resulted in increased CD8+ T cell infiltration and low numbers of highly activated NK cells in both tumor models. While this study focused on the lymphocytic compartment as the main effectors of IL-12 and IL-15 signaling, additional leukocyte populations most likely affect the therapeutic outcome. Accordingly, histological analyses revealed a mixed immune infiltrate, especially at tumor margins.

To gain insight into immune gene expression after treatment with MeVac vectors, we performed gene expression profiling and pathway analysis. Activation of NK, especially T cell effector responses, as well as diverse immune signaling pathways seemed to be stronger after treatment with MeVac P FmIL-12 in comparison to MeVac P FmIL-15. We reasoned that, aside from beneficial anti-tumor immunity, these effector cells might also mediate anti-viral immune responses, thereby limiting therapeutic efficacy. Interestingly, anti-viral signaling appeared to be upregulated after MeVac P FmIL-12, in comparison to MeVac P FmIL-15 treatment. Thus, premature viral clearance resulting from the activation of anti-viral immunity can largely be excluded as a cause of poor efficacy of MeVac P FmIL-15.

In summary, we conclude that, in comparison to MeVac FmIL-15, the efficacy of MeVac FmIL-12 was superior as a result of increased viral gene expression in vivo and enhanced anti-tumor immune effector responses. FmIL-12 is not necessarily intrinsically more suitable for immunovirotherapy. Rather, adjustments in MeVac FmIL-15 vector design to improve its replication kinetics, persistence, and bioavailability of the immunomodulator may enhance its therapeutic efficacy. A range of different immunomodulatory oncolytic vectors are currently considered as novel cancer therapeutics. It remains to be determined which agent is the most effective in which therapeutic setting. This study demonstrates that the efficacy of oncolytic immunotherapy results from the complex interplay of the viral vector, the tumor immune microenvironment, as well as local and systemic immune responses. The resulting therapeutic effects can hardly be predicted and must therefore be tested empirically in relevant model systems. The finding of superior efficacy of MeVac FmIL-12 in a syngeneic murine colorectal adenocarcinoma model motivated us to generate MeVac vectors encoding human IL-12 for future clinical translation. Vectors encoding FhIL-12 downstream of either the *P* or *H* open reading frame mediated efficient FhIL-12 secretion from infected cells and were not attenuated compared to parental MeVac. In line with previous reports on the susceptibility to measles oncolysis [17,57,58], human colorectal cancer cells were infected efficiently by oncolytic MeVac. Taken together, this study supports translational efforts towards a clinical trial with MeVac FhIL-12 against colorectal cancer.

## Supplementary Materials

The following are available online at https://www.mdpi.com/1999-4915/11/10/914/s1, Figure S1: Replication kinetics, cytokine expression and oncolytic activity mediated by MeVac FmIL-15 constructs. Figure S2: MC38cea tumors, HE-stain. Figure S3: Tumor volumes in individual mice. Figure S4: Protective anti-tumor immunity in long-term survivors. Figure S5: Ingenuity Pathway Analysis of NanoString data. Figure S6: Human colorectal cancer cells are susceptible to oncolytic measles virus. Supplementary Table S1: Histopathological analyses of MC38cea tumor samples. Supplementary files: Raw data of NanoString analysis.
