# Supplementary material for: Immunological Effects and Viral Gene Expression Determine the Efficacy of Oncolytic Measles Vaccines Encoding IL-12 or IL-15 Agonists
Source: Viruses. 2019 Oct 3;11(10):914. doi: 10.3390/v11100914 (PMC6832518; doi:10.3390/v11100914)
Supplement: Supplementary file 1 [file viruses-11-00914-s001.zip › Supplementary Figures.docx]

**Supplementary Figures**


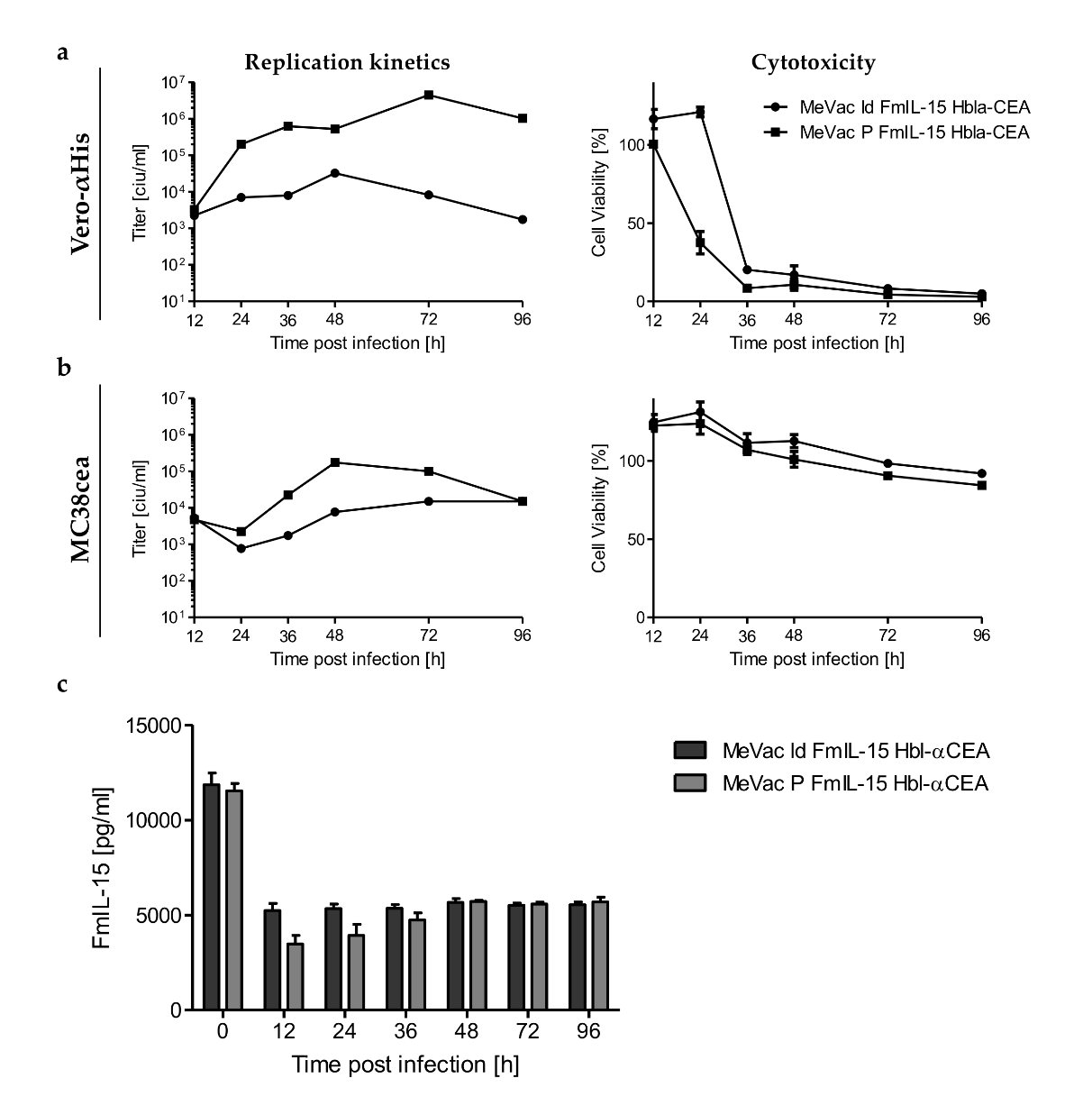


**Supplementary Figure 1.** Replication kinetics, cytokine expression and oncolytic activity mediated by MeVac FmIL-15 constructs. Vero-αHis and MC38cea cells were inoculated with indicated MeVac FmIL-15 variants at an MOI of 3. **(a)** Viral progeny at designated timepoints were quantified by titration assay. **(b)** Cell viability was determined using an XTT assay. **(c)** FmIL-15 in cell culture supernatants from MC38cea cells was measured by ELISA.


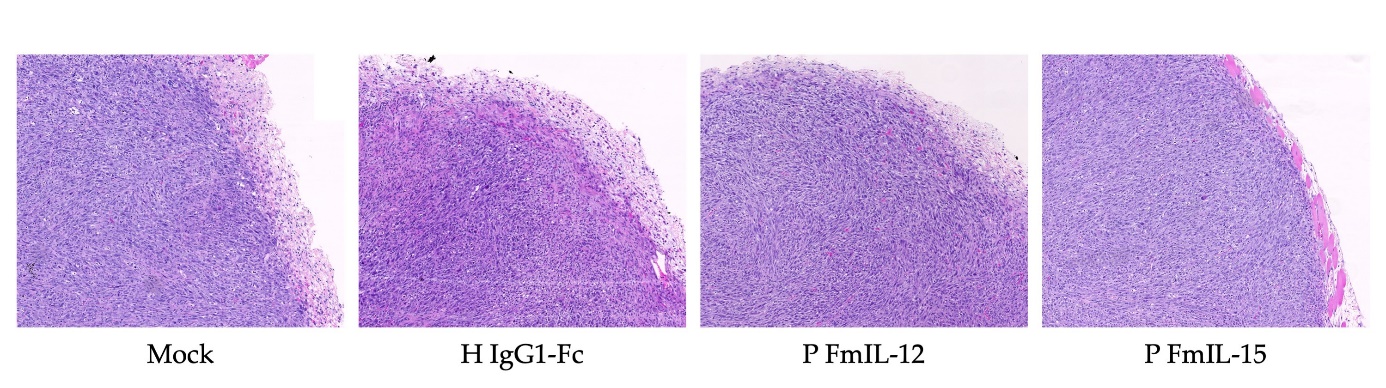


**Supplementary Figure 2.** MC38cea tumors, HE-stain. Tumors were explanted 24h after the last treatment with MeVac vectors, fixed with formalin and stained with hematoxylin/eosin. The images correspond to the following samples: mock “A”, H IgG-Fc “D”, P FmIL-12 “A”, P FmIL-15 “B” (compare sample IDs in Supplementary Table 1).


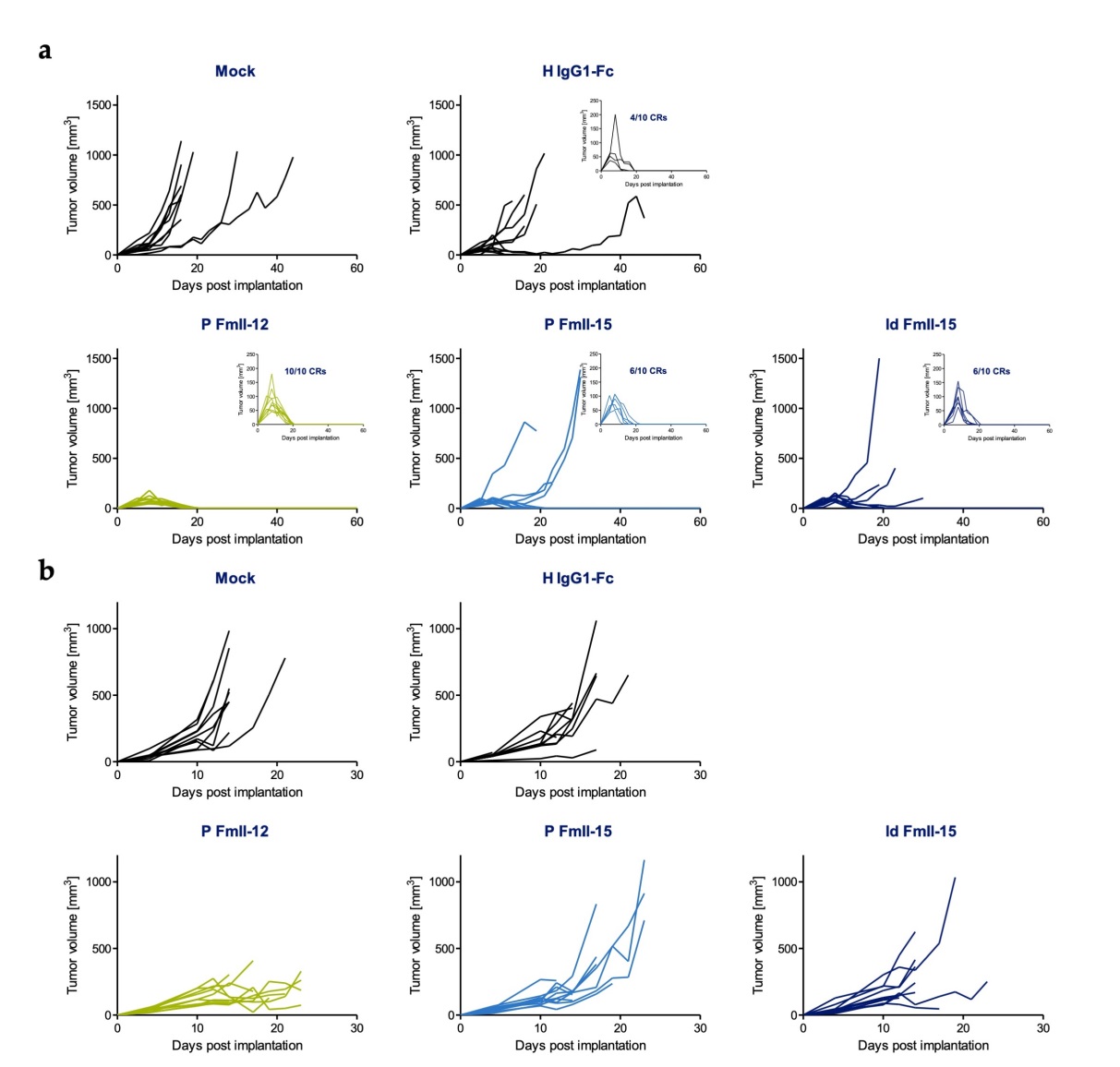


**Supplementary Figure 3.** Tumor volumes in individual mice. **(a)** MC38cea. **(b)** B16hCD46.


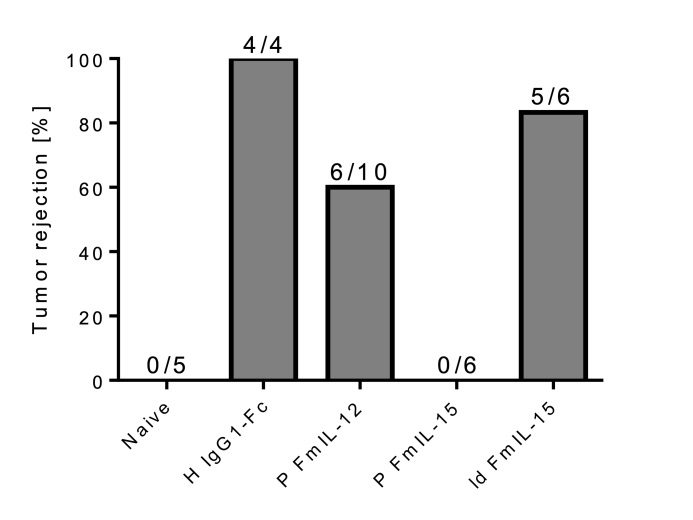


**Supplementary Figure 4.** Protective anti-tumor immunity in long-term survivors. Animals in complete remission after the MC38cea efficacy experiment were rechallenged with 1 x 10^5^ parental MC38 cells s.c. and monitored for tumor engraftment.


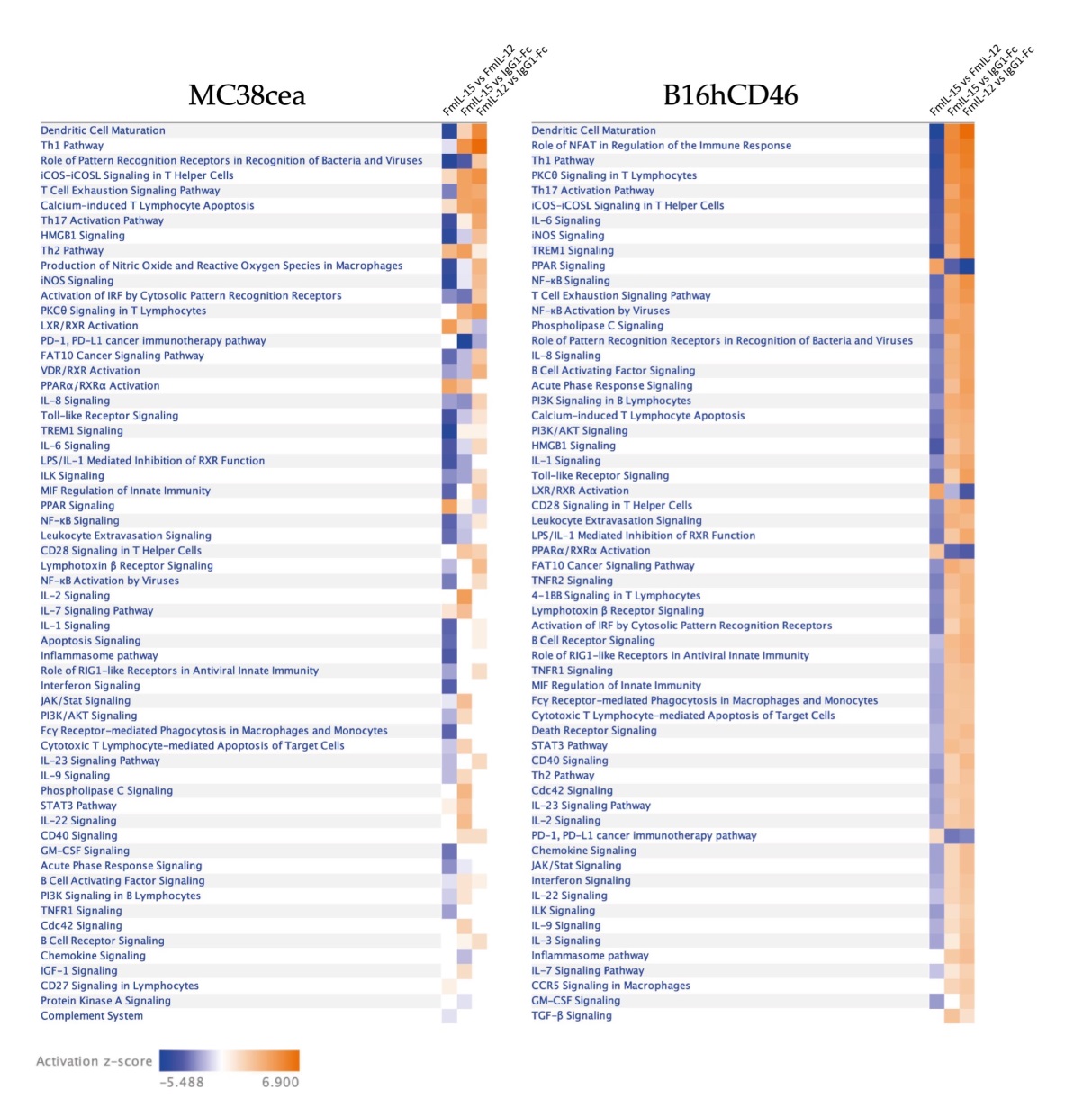


**Supplementary Figure 5.** Ingenuity Pathway Analysis of NanoString data. Ingenuity Pathway Analysis of differentially activated genes and pathways in indicated tumor models after treatment with different MeVac variants was performed based on gene expression data obtained from NanoString profiling using the Mouse Immunology Panel.


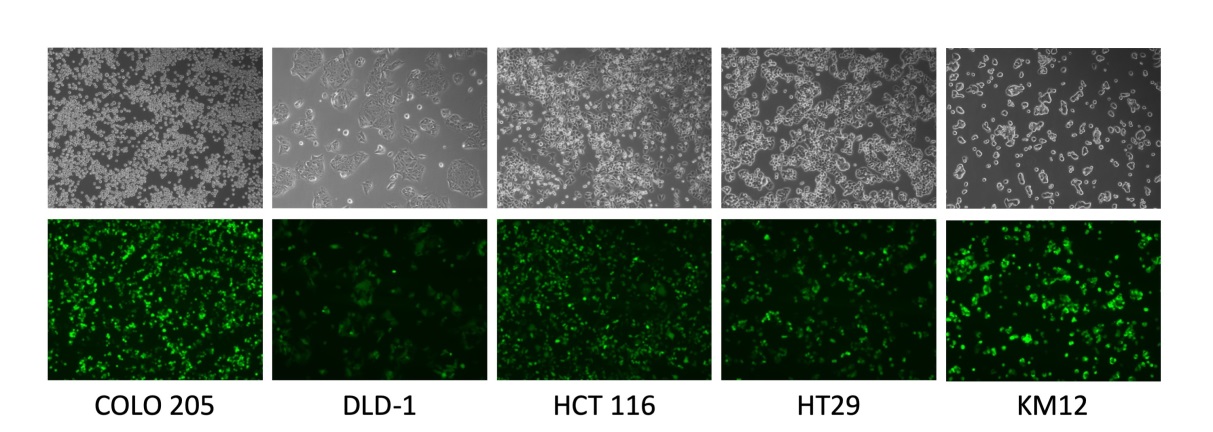


**Supplementary Figure 6.** Human colorectal cancer cells are susceptible to oncolytic measles virus. Indicated cell lines were left untreated (Mock; upper panels) or inoculated with MeVac ld EGFP at an MOI of 1 (lower panels). Images were acquired 24h after inoculation.
