# Supplementary material for: Immunological Effects and Viral Gene Expression Determine the Efficacy of Oncolytic Measles Vaccines Encoding IL-12 or IL-15 Agonists
Source: Viruses. 2019 Oct 3;11(10):914. doi: 10.3390/v11100914 (PMC6832518; doi:10.3390/v11100914)
Supplement: Supplementary file 1 [file viruses-11-00914-s001.zip › Supplementary Table 1.docx]

**Supplementary Table 1.** Histopathological analyses of MC38cea tumor samples. Four tumor samples from each treatment group were stained with hematoxylin-eosin and evaluated by a veterinary pathologist. Score: 0 – none, 1 – mild, 2 – moderate, 3 – severe; n.a.*; not assessable due to absence of peritumoral tissue; ** as far as assessable due to scarce peritumoral tissue.

| Treatment | ID | Necrosis | Intratumoral lymphocytes | Peritumoral inflammation | Comments |
| --- | --- | --- | --- | --- | --- |
| mock | A | 0 | 0 | 2 |  |
|  | B | 0 | 0 | n.a.* |  |
|  | C | 0 | 0 | 1** |  |
|  | D | 0 | 0 | 2 |  |
| H IgG-Fc | A | 0 | 0 | 2 |  |
|  | B | 0 | 0 | 2 |  |
|  | C | 0 | 0 | 2 |  |
|  | D | 0 | 0 | 3 |  |
| P FmIL-12 | A | 0 | 0 | 2 | acute death of many tumor cells |
|  | B | 0 | 0 | 2 |  |
|  | C | 0 | 0 | 3 | smaller cells and nuclei compared to other samples |
|  | D | 0 | 0 | 3 | smaller cells and nuclei compared to other samples |
| P FmIL-15 | A | 2 | 0 | n.a.* | acute widespread cell death |
|  | B | 0 | 1 | 1** |  |
|  | C | 0 | 0 | 3 | regional lymph node:  reactive hyperplasia |
|  | D | 0 | 0 | 1** |  |
